# Printed Circuit Board Defect Detection Based on Lightweight Deep Learning Fusion Model

**DOI:** 10.3390/s25247403

**Published:** 2025-12-05

**Authors:** Yuling Wang, Zhicheng Chen, Jie Wang, Peng Shang, Arcot Sowmya, Changming Sun

**Affiliations:** 1 School of Artificial Intelligence and Information Engineering, East China University of Technology, Nanchang 330013, China; jone3281@163.com (J.W.); 18298169924@163.com (P.S.); 2 School of Software, East China University of Technology, Nanchang 330013, China; 19047802507@163.com; 3 School of Computer Science and Engineering, University of New South Wales, Sydney, NSW 2052, Australia; a.sowmya@unsw.edu.au; 4 CSIRO Data61, Sydney, NSW 1710, Australia; changming.sun@csiro.au

**Keywords:** defect detection, PCB, lightweight deep learning model, feature fusion

## Abstract

Printed circuit boards (PCBs) are ubiquitous and essential electronic components. Tiny targets and high precision are the focus of PCB defect detection. This paper proposes an improved model focusing on tiny defect detection and model compression to achieve better performance in PCB defect detection. The improved model has a compact structure based on MobileNet v3 Small-CA and an image-cutting layer. Moreover, it applies an improved multi-scale fusion step with a location weighted mechanism to enhance representation performance. The proposed model outperforms state-of-the-art detection algorithms such as Faster R-CNN, EfficientDet, SSD, and YOLO v7, based on experimental results on a public synthetic PCB dataset. The proposed tiny object detection model has better performances on speed and detection accuracy, thereby benefitting the manufacturing industries associated with PCBs.

## 1. Introduction

Printed circuit boards (PCB) are the core element of electronic components, and during production they are vulnerable to defects such as missing holes, open circuits, spurs, mouse bites, short circuits, and spurious copper, presenting a variety of defect types, variable defect sizes and shapes, small defect areas, and defects that are similar to the background. Therefore, PCB defect detection is an important problem to address in the field.

Two popular PCB defect detection techniques used currently include radiographic test techniques and conventional image processing-based techniques. Radiographic tests (RT) use x-rays or γ-rays that can penetrate an object to detect defects in the internal structure. The process can reveal the nature, size and location of the defects, among other information. For real-time radiographic inspection of weld defects in spiral tubes, Zou et al. [1] suggested a Kalman filter-based automatic detection approach that offers some robustness in the case of unsteady detection speed. The current challenges that need to be addressed are, however, increasing the detection accuracy and efficiency, and decreasing the detection cost. Certain detection approaches based on conventional image processing to solve the aforementioned issues have also been developed. They primarily classify images using histograms of oriented gradients (HOG) [2] and scale invariant feature transform (SIFT) [3]. Although the defect detection method using HOG features has high accuracy and speed, HOG features are not scale invariant, and the detection framework is essentially a global rigid template model, which requires global matching of the entire object and has a high computational cost. Khan et al. [4] summarised the performance of SIFT on several classification datasets and proposed three shorter SIFT descriptors. The computational cost can be successfully reduced using the suggested methods. Ong et al. [5] proposed the SEConv-YOLO model, which incorporates a lightweight SEConv feature extraction module, a WRSPP feature fusion neck, and an N-CIoU loss function to significantly improve the accuracy and efficiency of defect detection in PCBs and PCB wiring.

Wang et al. [6] proposed the HSA-RTDETR method for PCB defect detection, incorporating a new R18-Faster-EMA backbone, an improved AIFI module, a HS-PAN, and an MPDIoU + NWD loss function. This approach significantly improves detection accuracy for small defects and speeds up model convergence. Despite this, there is still much space for improvement in detection efficiency. Especially for complex and diverse PCB defects, the detection accuracy needs to be much improved.

In recent years, with the advancement of deep learning, defect detection based on deep learning technology is one of the current research foci [7,8,9]. Faster R-CNN [10,11] for two-stage defect detection and YOLO [10] for one-stage defect detection are the two primary algorithms used by current deep learning-based PCB defect detection approaches. Among them, Faster R-CNN has a high detection accuracy and a fast detection speed, because it uses anchor frames corresponding to the original image for obtaining the feature map [12]. Nevertheless, the anchor frames are subjected to several downsampling procedures and cover a larger portion of the original image, which makes it difficult for Faster R-CNN to effectively detect small PCB defects. The single-stage detection algorithm YOLO v7 can detect defects at different scales [13] and has good performance in detecting tiny defects, which helps overcome the issue of misdetection and missed detection on tiny defects. The YOLO v7 network involves a large number of parameters and calculations, making it challenging to deploy the network on lightweight devices.

In order to further decrease the number of parameters and computational cost of the network to facilitate deployment on lightweight devices [14], improve detection accuracy, and avoid misdetection of minor defects, this paper proposes a PCB defect detection strategy based on a lightweight deep learning fusion model (LDLFModel). The model uses the YOLO v7 architecture as a reference and a lightweight network with the location attention mechanism called MobileNet v3 Small-CA as the backbone network, which has fewer parameters and lower computational cost. The location attention mechanism is applied in the multi-scale feature fusion module, and weighted feature fusion is performed on the feature map to improve the defect detection performance. Meanwhile, EIoU-loss is employed as the target frame regression loss to make the localisation on the prediction box more accurate. Finally, the detection module has been upgraded to include an image-cutting layer and a restoration layer to address the issue of minor defect misdetection [15]. The result of defect detection on PCBs using the LDLFModel is significantly better than using YOLO v7 alone.

## 2. YOLO v7 Network Model

YOLO is currently a detection method that has advantages in detection speed, and it has been applied in many areas and can meet real-time requirements. The YOLOs have evolved from the first to the tenth versions, and YOLO v7 is more widely used in practical projects.

The network structure of YOLO v7 includes the following components: input, backbone, and head. An input image needs to be preprocessed, and data augmentation is adopted. Four random images are read from the input dataset, parts of the four images are taken for splicing, and the spliced images are used for data augmentation via random scaling, random flipping, random clipping, random perspective projection, and other operations so as to enrich the background of the defects and indirectly improve batch size, which can reduce the challenges of training. Meanwhile, YOLO v7 uses an adaptive anchor box to obtain the best anchor box parameter in the training set during training. Then, the input images are adjusted to an appropriate size according to the input model size, and they are sent to the detection network.

In the backbone, CSPDarkNet, which adds the E-ELAN structure and the MP structure, is employed to extract the image features. Among them, the function of the E-ELAN structure is to improve the learning ability of the model, and the role of the MP structure is to improve the feature fusion ability of the model. Based on an input image with 960 × 960 pixels to be sent to CSPDarkNet, the three RGB channels undergo a Focus operation to obtain a feature map of size 480 × 480 × 12. The feature maps are used for a series of convolution operations, and then three effective feature layers of size 120 × 120, 60 × 60, and 30 × 30 are obtained through the E-ELAN network and the MP structure.

Subsequently, these three effective feature layers are input into the head part, which utilises a feature pyramid network (FPN) and a path aggregation network (PAN) to fuse features. FPN, with a top-down approach, fuses the features extracted from the backbone network to convey deep semantic information. PAN conveys the location information using a bottom-up approach. In addition, the SPPCSPC structure, the E-ELAN structure, the MP structure, and RepConv are added to the head section. Among them, the role of SPPCSPC is to further expand the receptive field to improve the detection accuracy of the model. The role of RepConv is to improve the inference speed of the model. Then, three enhanced feature layers are obtained. Finally, the prediction component, namely the YOLO head, adjusts the channels of these enhanced feature layers to obtain three feature-cubes of sizes 120 × 120 × 33, 60 × 60 × 33, and 30 × 30 × 33. Then, the position of the centre point, the width and height of the target box, the confidence values, and the target categories of the feature layers are predicted.

## 3. Proposed LDLFModel

**Figure 1:**
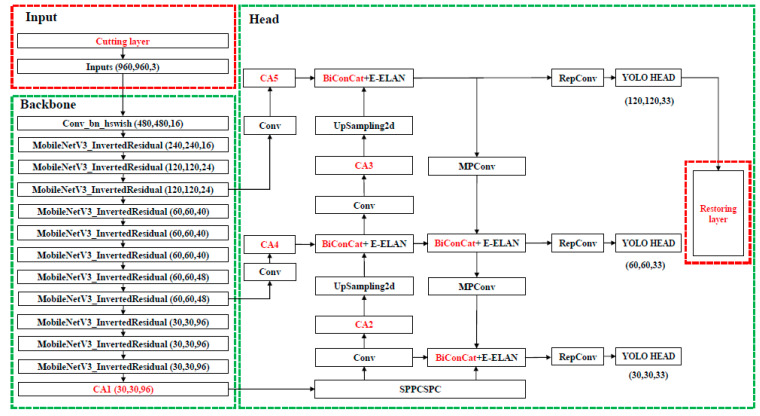
The architecture of the LDLFModel network.

In order to better meet the deployment requirement on lightweight devices and improve detection performance in PCB defect detection, this paper proposes a lightweight deep learning fusion model (LDLFModel). While our approach uses the YOLO v7 framework as a reference, our primary contribution lies in the novel integration and customized optimization of its components to address the specific challenges of PCB defect detection, namely model lightness, tiny defect misdetection, and feature fusion efficiency. Firstly, beyond merely adopting a lightweight backbone, our technical contribution involves the design of a specialized backbone network, MobileNet v3 Small-CA. This is achieved by strategically embedding the coordinate attention (CA) mechanism into the MobileNet v3 Small architecture. We conducted extensive ablation studies to determine the optimal number and placement of CA modules, creating a backbone that is not only lightweight but also possesses enhanced location-aware capabilities crucial for pinpointing small defects. Secondly, we introduce a customized multi-scale feature fusion scheme. Instead of a simple feature addition in the head component, we implement a learnable weighted fusion mechanism and strategically insert CA modules into the fusion paths. This design, which assigns adaptive weights to features of different resolutions and reinforces spatial information during fusion, represents a key enhancement aimed at significantly boosting feature representation for multi-scale defects. Thirdly, to tackle the persistent problem of tiny defect misdetection, we propose a dedicated image-cutting layer. This module systematically divides high-resolution input images into overlapping patches, processes them independently, and then seamlessly reassembles the results. This pre-detection strategy serves as our focused solution to prevent the loss of fine-grained details, ensuring that even the smallest defects are captured effectively. Finally, the target box regression loss is replaced with EIoU-loss to improve localization accuracy. Together, these key innovations—the optimized MobileNet v3 Small-CA backbone, the weighted and attention-enhanced feature fusion, and the innovative image-cutting layer—constitute the core of the proposed LDLFModel, enabling high precision in detecting PCB defects of all sizes while maintaining a compact and efficient model suitable for lightweight devices. The overall LDLFModel network architecture is shown in Figure 1.

### 3.1. Backbone Adopts MobileNet v3 Small-CA

The backbone adopted by YOLO v7 is the Improved CSPDark-Net. Although the backbone network has good performance on feature extraction, there are problems of excessive parameters and computation, which makes it difficult to deploy the overall model on low performance devices. Designing efficient and smaller lightweight models is undoubtedly a great approach. Therefore, the LDLFModel adopts the lightweight network MobileNet v3 Small as the backbone to construct a feature extraction network based on the location attention mechanism.

MobileNet v3 Small inherits the deep separable convolutions [16] and the inverted residual structure with linear bottlenecks [17], and adds a network search structure, a squeeze and excitation (SE) module [18], and activation functions Hard-sigmoid and Hard-swish which are composed of Conv2D, Inverted-bottleneck, Inverted-bottleneck-shortcut, and pooling. The Inverted-bottleneck consists of two 1 × 1 convolutions and a 3 × 3 deep separable convolution. In the latter, the feature map of each layer is convolved by using the appropriate convolution kernel separately, considerably reducing the number of parameters and computational complexity, while also enhancing the convolution speed. The Inverted-bottleneck-shortcut contains a shortcut in addition to Inverted-bottleneck. The shortcut can fuse the output feature map of the convolution layer and the input information so as to prevent gradient explosion.

**Figure 2:**
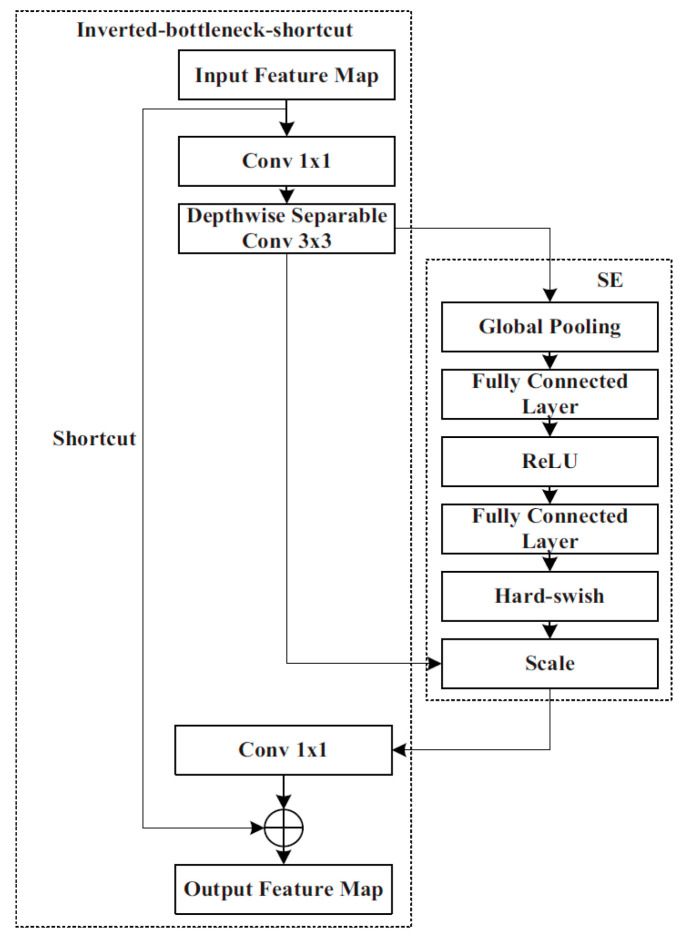
MobileNet v3-SE.

**Figure 3:**
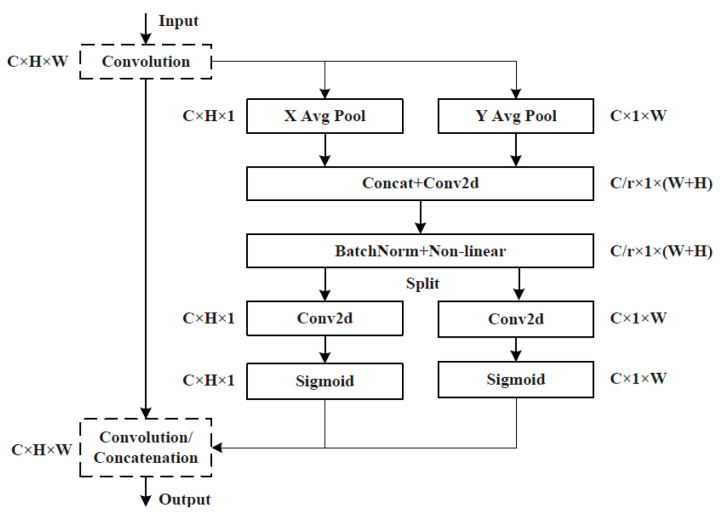
The CA attention mechanism in the network structure.

MobileNet v3 Small also contains SE attention modules that include two operations: ‘*Squeeze*’ and ‘*Excitation*’ (SE), which are used for embedding global information and weighting relationships of adaptive channels, respectively. By means of global average pooling, ‘*Squeeze*’ compresses W × H features of the feature map in each channel into a group of real numbers for obtaining global information. ‘*Excitation*’ performs dimension reduction, dimension addition, and activation processing on the group of real numbers. As a result, a group of new real numbers between 0 and 1 is obtained. Finally, these numbers are multiplied by the value of each pixel of the feature map in the corresponding channel to realise the attention weighting of the feature map and complete the capture of the weight of each channel. MobileNet v3-SE is shown in Figure 2. A CA attention module is additionally integrated into the last layer of the LDLFModel backbone network to improve the capability of the backbone network feature description, which is sensitive to location and orientation, and to make up for the SE module’s lack of location information. The CA attention module is displayed in Figure 3.

### 3.2. Changing the Target Box Regression Loss Function to EIoU-Loss

**Figure 4:**
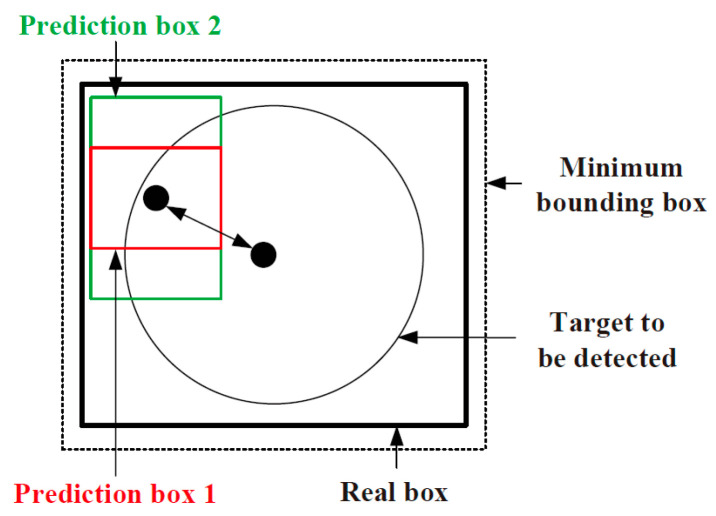
Relationship between the prediction box and the real box.

The YOLO v7 network employs the *CIoU-loss* target box loss function, and the correlation between the prediction box and the real box is shown in Figure 4. The aspect ratio, overlapped area, and centre point distance of the boundary box regression are taken into account by the target box loss function. Nevertheless, the formula only reflects the difference in aspect ratio, and does not reveal the real differences between widths, heights, and their confidences. Therefore, it hinders the effective optimisation of model similarity. For this problem, LDLFModel uses a better target box loss function, EIoU-loss, on the basis of *CIoU-loss*, which utilises the real differences in height and width instead of aspect ratio, and introduces focal loss to optimise the sample out-of-balance issue in the bounding box regression tasks to obtain better anchor frames, thereby improving the accuracy of defect detection.

According to *CIoU-loss*, the centre point distance of the prediction box and the real box is denoted as *ρ*(*b*, *b^gt^*). The difference of their widths is denoted as *ρ*(*w*, *w^gt^*). The difference of their heights is denoted as *ρ*(*h*, *h^gt^*). The diagonal length of the smallest bounding rectangle, including the prediction box and the real box, is denoted as *c*. The width and height of the smallest bounding rectangle are denoted as *C_w_* and *C_h_* respectively. The intersection ratio of prediction box A and real box B is:

(1) $$IoU=\frac{\left|A\cap B\right|}{\left|A\cup B\right|}$$

The final target box loss function is:

(2) $$\zeta_{EIoU}=1-IoU+\frac{\rho^{2}(b,b^{gt})}{c^{2}}+\frac{\rho^{2}(w,w^{gt})}{C_{w}^{2}}+\frac{\rho^{2}(h,h^{gt})}{C_{h}^{2}}$$

### 3.3. Weighted Feature Fusion Optimization

The FPN of the head component of YOLO v7 adopts the top-down approach to fuse with the features extracted from the backbone network to transfer the deep semantic information, while the PAN uses the bottom-up approach to transfer position information to construct the feature fusion network. However, in many models, features from different levels or sources are often fused through simple concatenation. To address this limitation, our LDLFModel introduces a custom fusion module named BiconCat (as shown in Figure 1). Inspired by BiFPN, BiconCat moves beyond simple concatenation by integrating a fast normalized fusion mechanism. This mechanism adaptively weights multi-scale features from bidirectional paths, forming a crucial part of our enhanced feature fusion strategy. Although different input features are fused, they are just simply added. However, due to the different resolutions of these various input features, their contributions to the fused output features are also different. In order to further enhance the effect of feature fusion via the Head of YOLO v7, the LDLFModel adds weights to each feature fusion layer and quickly adopts a normalised fusion [19] to realise the weighted fusion of features. Considering that each 1 × 1 convolutional layer in the head section can obtain a greater degree of global information, the CA attention mechanism is added after the convolution layers in the head component. The results O of the fast normalised fusion process are as follows:

(3) $$O=\sum_{i}\frac{w_{i}}{lr+\sum_{j}w_{j}}\cdot I_{i},\;\mathrm{and}\;i,j\in \left\{1,2,\ldots ,13\right\}$$

where *w_i_* represents the learning weight corresponding to the *i*-th input feature *I_i_* and *lr* equals 0.0001 to ensure that the denominator is not 0. The fusion result *P_x_^o^^ut^* in the *x*-level feature layer can be expressed by the following formula:

(4) $$P_{x}^{out}=Conv\left(\frac{w_{1}\cdot P_{x}^{in}+w_{2}\cdot Resize(P_{x+1}^{in})}{w_{1}+w_{2}+\in }\right)$$

where ‘*Resize*’ refers to the up or down sampling according to the size of the feature map, ‘*Conv*’ refers to the convolution operation on features, *w*_1_ and *w*_2_ represent the weights, *P_x_^o^^ut^* represents the output feature after the fusion at the *x*-level feature layer, and *P_x_^in^* represents the input feature at the x-level feature layer. A small constant ϵ= 0.0001 is introduced to prevent division by zero in the denominator.

By integrating fast normalized feature fusion into the head of the LDLFModel, this approach effectively addresses the challenge of poor fusion among feature layers of different resolutions, enhancing the overall feature fusion performance. Compared with the simple eigenvalue connection, the accuracy of the fast normalised fusion method is improved by 2.4%, the recall rate is improved by 7.3%, and the mAP is improved by 4.3%. The advantages of fast normalised fusion are obvious.

### 3.4. Adding a Image-Cutting Layer to the Detection Module

High resolution images obtained by modern imaging technologies can ensure that even minor defects in PCB images are also informative enough, which is more conducive to the detection of minor defects. However, if a high resolution image is directly input to the network for detection, the subtle image details may be lost due to the resizing operations, and then the information about the minor defect features will be insufficient, resulting in misdetection of minor defects. The input image size in the LDLFModel without the image-cutting layer is 960 × 960 pixels. By down-sampling, the output sizes of feature maps are 120 × 120, 60 × 60, and 30 × 30. The 120 × 120 feature map is mainly responsible for detecting tiny defects. The receptive field of each grid is 960/120 = 8. Assuming that the high resolution PCB image has a resolution of *M* × *N*, then if the width and height of tiny defects in the original image are less than 120 pixels, the tiny defects will become too small and may be missed. Certainly, if the input image resolution is high, a device with a higher computation power is required.

**Figure 5:**
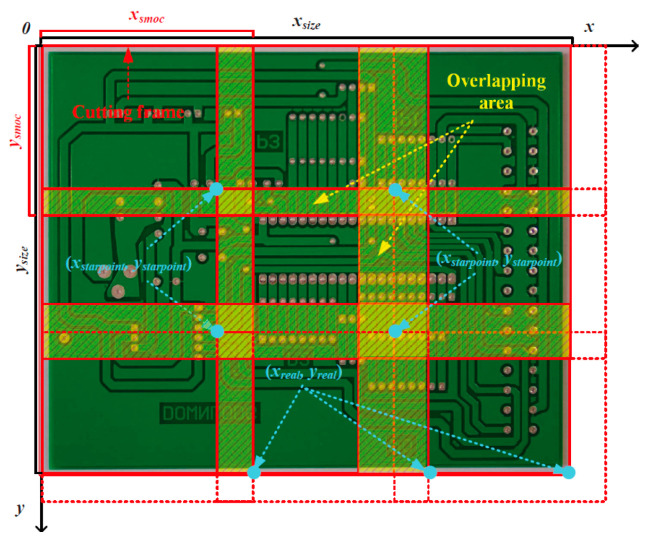
The cutting process.

In this study, small defects are defined as those with bounding box dimensions between 30 × 30 and 60 × 60 pixels in the original high-resolution PCB images (2544 × 2156 pixels). This criterion is consistent with common definitions of small objects in detection tasks such as traffic monitoring. To solve the issue of missing tiny defects, the LDLFModel adds an image-cutting layer to the detection network. The cutting process is shown in Figure 5. First, a cutting factor *mul* is set, and *mul* is used to control the number of small images to be obtained. With *mul* = 1 the image-cutting layer can be turned off, and *mul* > 1 indicates that the image-cutting layer is turned on. The relationship between the number of small images obtained and *mul* is defined as follows:

(5) $$n=wul^{2}$$

Next, the width *x_size_* and height *y_size_* of the minimum cutting frame is obtained. Then the width *x_smoc_* and height *y_smoc_* of the minimum cutting frame is calculated:

(6) $$x_{smoc}=\frac{x_{size}}{mul}\times 1.2$$

(7) $$y_{smoc}=\frac{y_{size}}{mul}\times 1.2$$

To ensure that any PCB tiny defects to be detected are not cut in half, a 20% overlap area between adjacent cut images is set. Then the cutting step is set to 64 pixels. Afterwards, the starting coordinate of each minimum division frame (*x_startpoint_*, *y_startpoint_*) is calculated:

(8) $$x_{starpoint}=i\times \frac{x_{size}}{mul}(if\;i<mul)$$

(9) $$y_{starpoint}=j\times \frac{y_{size}}{mul}(if\;j<mul)$$

where *i* and *j* are natural numbers. Then the relative coordinates of each minimum division frame (*x_real_*, *y_real_*) are calculated:

(10) $$x_{real}=min(x_{starpoint}+x_{smoc},x_{size})$$

(11) $$y_{real}=min(y_{starpoint}+y_{smoc},y_{size})$$

According to the relationship among the starting coordinates of each minimum division box, each relative coordinate, and the step size, it can be determined whether the image is completely cut at the current moving step size. If it cannot be completed, the previous cutting box is aligned with the right or bottom border of the image. Finally, the obtained small image is sent to the detection module for detection. Visualisation of the detection results can be carried out. After all the small images are obtained, the restoration layer will receive the small images with the results of the detection frames. The small images will be reassembled according to the coordinates of the lower right corner (*x_smoc_*, *y_smoc_*) of each minimum cut frame to restore the original large images. To adapt the input size for detecting small defects, the original high-resolution image (2544 × 2156 pixels) is divided into a 3 × 3 grid of sub-images. A 20% overlap, relative to the original image’s width and height, is applied between adjacent sub-images to avoid missing defects at the boundaries. Each resulting sub-image is then center-padded and resized to a fixed size of 960 × 960 pixels, ensuring a consistent and distortion-free input for the detection network. Finally, non-maximum suppression is performed, and any redundant frames are removed on the restored PCB image with the detection results.

## 4. Experiments and Analysis

This work used a Linux system and the PyTorch deep learning framework. The lectotype of GPU was provided by AutoDL (https://www.autodl.com). The configuration of the server processor was as follows: the CPU is 9-core Intel (R) Xeon (R) Silver 4210R CPU at 2.40 GHz and the memory is 256 GB. The model of the graphics processor is NVIDIA GeForce RTX 3080 and the graphics memory is 10 GB. The software environment is PyTorch 1.9.0, Python 3.8, and CUDA 11.1.

### 4.1. Evaluation Metrics

In defect detection, the two tasks, namely positioning and classification, need to be completed, which are measured by mean average precision (mAP). This is the average value of average precision (AP) on various defects. AP is determined by precision and recall in the classification results. Precision refers to the ratio of real positive samples for prediction. Recall indicates the proportion of positive samples with correct prediction in the actual positive samples.

(12) $$mAP=\frac{\sum_{i=1}^{n}AP(i)}{n}$$

(13) $$AP=\int_{0}^{1}P(R)dR=\sum_{k=1}^{N}P(k)\Delta R(k)$$

(14) $$Presion=\frac{TP}{TP+FP}$$

(15) $$Recall=\frac{TP}{TP+FN}$$

where *TP*, *FP* and *FN* represent true positive (both detection and ground truth are positive), false positive (detection is positive and ground truth is negative) and false negative (detection is negative and ground truth is positive), respectively. *n* indicates the number of categories of the defects to be detected. *N* is defined as the number of all images in the test set. At the same time, to further demonstrate the advantages of the LDLFModel using a lightweight backbone network, the LDLFModel was compared to other models in terms of model size, number of parameters, and computational cost.

### 4.2. PCB Dataset and Data Augmentation

**Figure 6:**
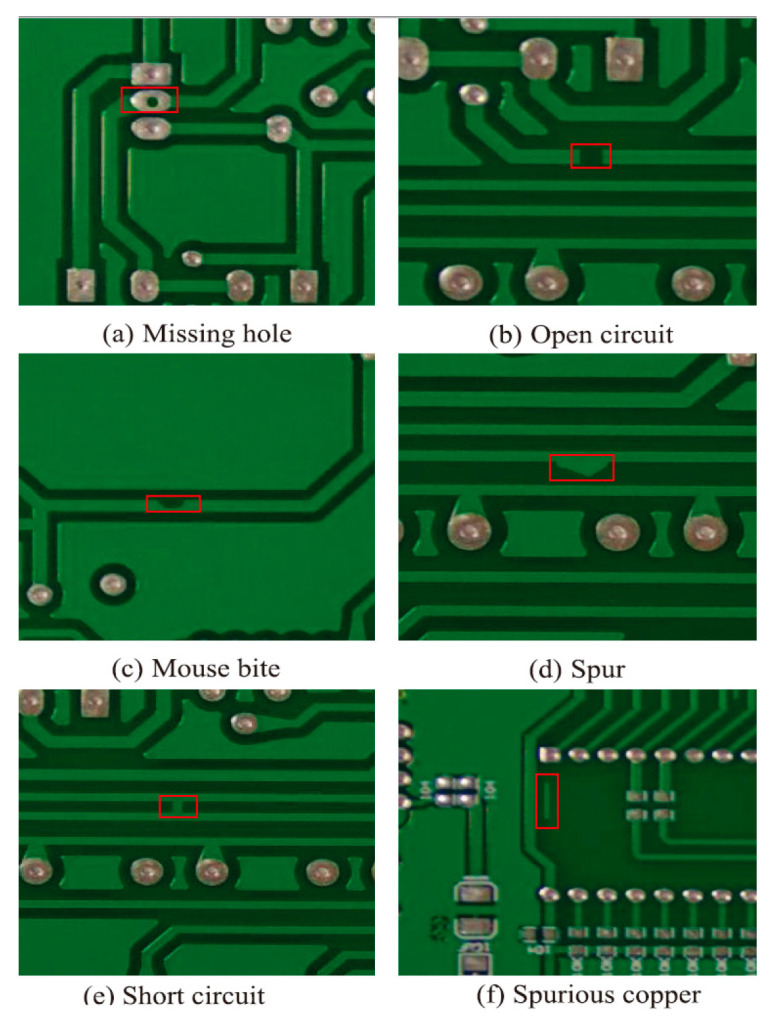
Samples of each type in the dataset.

The PCB dataset used in this paper is derived from the Open Laboratory of Intelligent Robotics at Peking University, China. The dataset contains six types of defects, i.e., missing hole, open circuit, mouse bite, spur, short circuit, and spurious copper. Data augmentation is performed to expand the sample size in this work by random flipping, random brightness change, and random scaling of the PCB images in the original dataset. The augmented dataset contains 10,800 PCB images with defects. The information on the augmented dataset is shown in Table 1. The dataset is randomly divided into training set and test sets according to a ratio of 9:1. Examples of PCB images are depicted Figure 6.

Using data augmentation, the proposed model is enhanced for robustness. When the numbers of parameters are the same, the changes in precision, recall, and mAP are compared through experiments on the datasets before and after data augmentation. We observe a significant (about 5%) increase in recall, and an obvious increase (about 2%) in precision and mAP. Therefore, data augmentation is an important factor in the model’s performance.

### 4.3. Lightweight Backbone Network and Epochs

In the case of only changing the backbone network, where the model is trained using the data augmented dataset, the precision, recall, mAP, the number of parameters, computational cost, and the size of the trained model are compared when PPLCNet, ShuffleNet-v2, and Efficient lite are used as the lightweight backbone. The results in Table 2 show that MobileNet v3 Small has the best overall performance compared to PP-LCNet, ShuffleNet-v2, and Efficient lite, and is more suitable as the backbone of the LDLFModel.

The number of iterations will greatly affect the recognition results of the model. We conducted comparative experiments on different numbers of iterations on the augmentation dataset. The samples are randomly divided into training set and test set at a ratio of 9:1. The results in Table 3 show that precision, recall and mAP have been improved to a certain extent. When the number of iterations is set to 300, the precision, recall, and mAP reach the maximum upper limit, and then the final number of iterations is set to 300 epochs in the following experiments.

### 4.4. Comparative Experiment and Result Analysis

The LDLFModel proposed in this paper is compared with various models in terms of the number of parameters, model size, and inference time, as shown in Table 4. The baseline model YOLO v7 has a model size of 74.8 MB, 36.49 million parameters, and an inference time of 18.3 ms. Simply replacing its backbone CSPDarkNet with the lightweight MobileNet v3 Small reduces the model size to 10.4 MB, the number of parameters to 7.2 million, and the inference time to 13.5 ms. Compared with other advanced models, C3F-SimAM-YOLO has a model size of 11.4 MB and 5.5 million parameters; SCF-YOLO has 9.7 MB and 6.1 million parameters; and YOLOv8-PCB has 5.6 MB and 5.9 million parameters. The proposed LDLFModel further reduces the model size to 5.4 MB and has 5.6 million parameters, achieving the best performance with an inference time of 12.7 ms while maintaining competitiveness.

The comparative experiment used the augmented dataset described in Section 4.1, and the comparison of the model’s computational cost and detection performance is shown in Table 5. The benchmark model YOLO v7 has a computational cost of 16.4 GFLOPs, with an accuracy, recall, and mAP of 93.6%, 93.2%, and 94.2%, respectively. After replacing its backbone network with MobileNet v3 Small, the computational cost dropped to 6.3 GFLOPs. Compared with other advanced models, C3F-SimAM-YOLO achieves an accuracy of 98.0%, recall of 95.4%, and mAP of 97.5% at a computational cost of 12.6 GFLOPs; SCF-YOLO achieves an accuracy of 98.7%, recall of 96.3%, and mAP of 98.3% at 3.4 GFLOPs; and YOLOv8-PCB achieves an accuracy of 94.7%, recall of 94.0%, and mAP of 96.1% at 7.1 GFLOPs. Experimental results show that the computational cost of the LDLFModel in this study is significantly reduced to 1.6 GFLOPs. Compared with existing state-of-the-art models, it is 11.0G lower than C3F-SimAM-YOLO, 1.8G lower than SCF-YOLO, 5.5G lower than YOLOv8-PCB, 14.8 GFLOPs lower than YOLO v7, and 4.7 GFLOPs lower than MobileNet v3 Small-YOLO v7. In terms of detection performance, the LDLFModel achieves an accuracy, recall, and mAP of 99.4%, 97.6%, and 99.0%, respectively, demonstrating comprehensive advantages. Compared with SCF-YOLO, they increase by 0.7%, 1.3%, and 0.7%, respectively; compared with C3F-SimAM-YOLO, the three metrics increase by 1.4%, 2.2%, and 1.5%, respectively; compared with YOLOv8-PCB, the improvements are more significant, reaching 4.7%, 3.6%, and 2.9%, respectively. Additionally, compared with YOLO v7, the metrics increase by 5.8%, 4.4%, and 4.8%, and compared with MobileNet v3 Small-YOLO v7, they improve by 0.7%, 2.2%, and 1.6%, respectively. Notably, while the LDLFModel significantly reduces computational cost, it outperforms all other comparison models across all performance metrics, excelling not only in computational efficiency but also leading comprehensively in detection accuracy, demonstrating outstanding overall performance and achieving an optimal balance between accuracy and efficiency.

**Figure 7:**
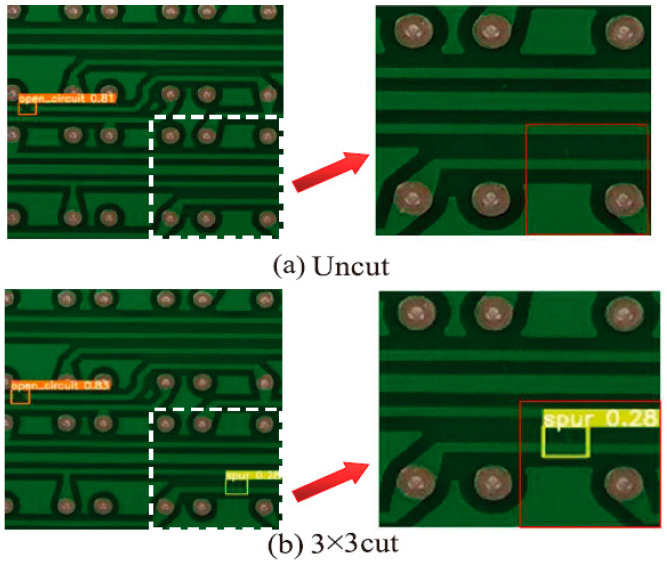
Comparison of detection effect between uncut and 3 × 3 cut.

In addition, in order to further demonstrate that the image-cutting layer of the LDLFModel has a certain effect on the problem of misdetection and missed detection of tiny defects, this paper compares the detection results of YOLO v7 and the LDLFModel with a 3 × 3 image-cutting layer. The comparisons are shown in Figure 7. It can be seen that the LDLFModel can detect tiny defects, which can effectively solve the problems of misdetection of small PCB defects. In conclusion, the LDLFModel greatly reduces the number of parameters and computational cost while maintaining high detection accuracy. It also effectively solves the problem of tiny defect misdetection and missed detection, making it more suited for industrial PCB defect detection requirements.

**Figure 8:**
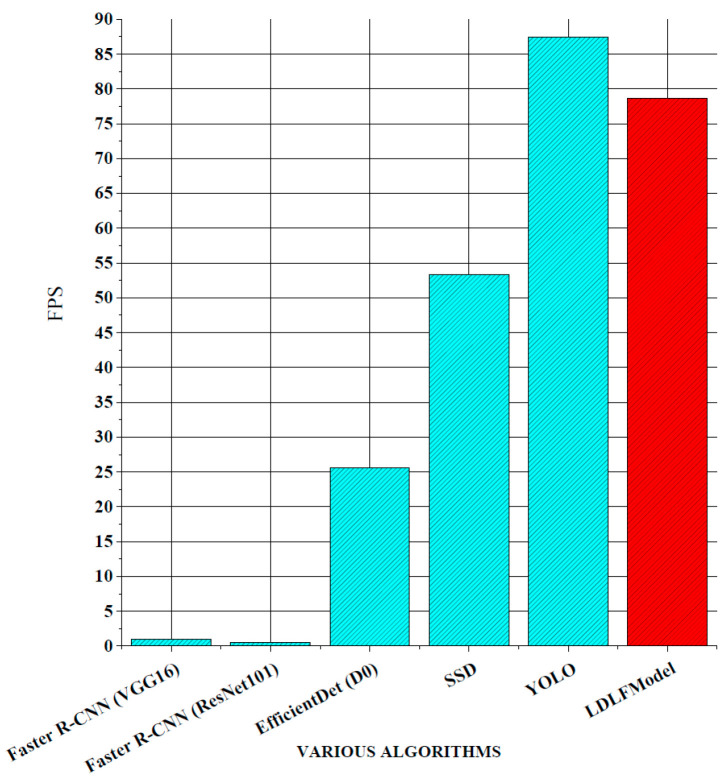
FPS comparison.

**Figure 9:**
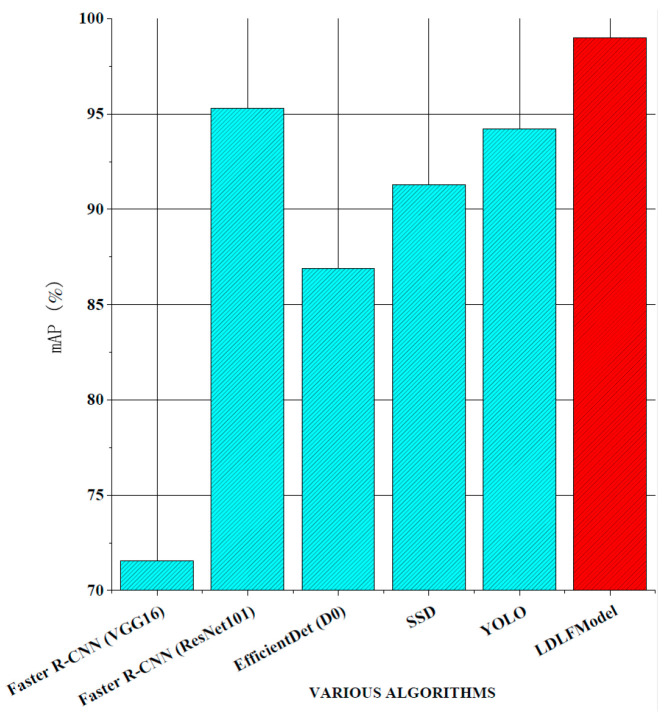
mAP comparisons.

**Figure 10:**
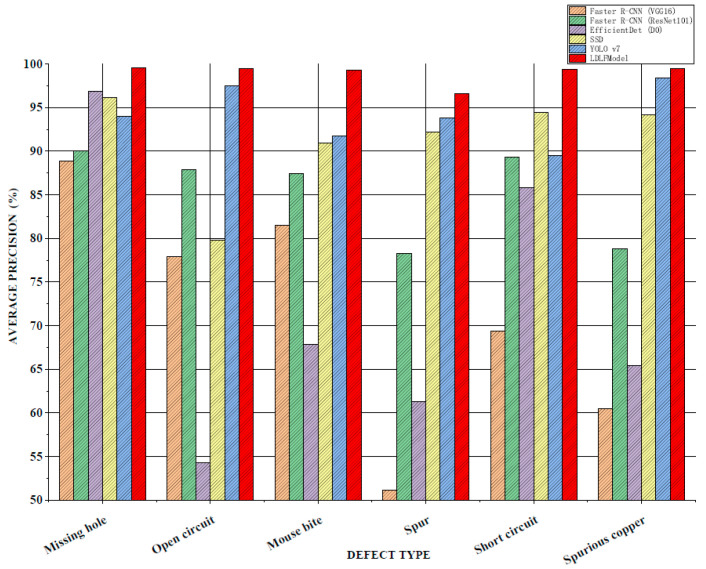
AP comparison of the detection of six types of defects.

For verifying the performance of the LDLFModel, the proposed model was compared to several other mainstream algorithms. The classic two-stage algorithms are Faster R-CNN (VGG16) and Faster R-CNN (ResNet101), which use VGG16 and ResNet101 [23] as the backbone networks, respectively. The classic one-stage algorithms are Efficient-Det (D0) [24], single shot multibox detector (SSD) [25], YOLO v7, and LDLFModel. The frames per second (FPS) and mAP of various algorithms and the AP of each type of PCB defect are shown in Figure 8, Figure 9 and Figure 10.

As shown in Figure 8, the LDLFModel without the image-cutting layer achieves 78.73 FPS, outperforming Faster R-CNN (VGG16) at 77.68 FPS, Faster R-CNN (ResNet101) at 78.14 FPS, EfficientDet (D0) at 53.11 FPS, and SSD at 25.31 FPS. Its speed is also comparable to that of YOLO v7, indicating the LDLFModel’s superior detection speed. Figure 9 demonstrates that the LDLFModel reaches a maximum mAP of 99.0%, exceeding the performance of Faster R-CNN (VGG16), EfficientDet (D0), and SSD by 27.45%, 12.1%, and 7.72%, respectively. It also outperforms YOLO v7 and Faster R-CNN (ResNet101) with mAP improvements of 4.8% and 3.7%. The results demonstrate that the LDLFModel delivers both high detection speed and strong accuracy, making it a reliable choice for defect detection.

For six types of PCB defects, the APs of various algorithms are shown in Figure 10. It can be seen that compared to the Faster R-CNN (VGG16), Faster R-CNN (ResNet101), and EfficientDet (D0) algorithms, the APs of the LDLFModel on various defects are much higher than that of the other algorithms. Compared to the APs from the Faster R-CNN (VGG16) algorithm on the stray and spurious copper defects, the APs from the LDLFModel is 45.47% and 39.05% higher respectively. Compared to the APs on the six types of defects from YOLO v7, the proposed model has improved results. The above results show that the LDLFModel can achieve the detection requirements of multiple types of defects simultaneously, and high accuracy for six types of defects of PCBs.

### 4.5. Ablation Studies

#### 4.5.1. Regression Loss Functions

Bhattacharya and Cloutier [26] compared the performance of CIoU, DIoU, GIoU, and IoU. They found that CIoU outperformed the other loss functions. He et al. [27] presented a generalization α-IoU loss that remarkably surpasses the IoU-based losses, and pointed out that better results will be achieved with α = 3. We conducted experiments to compare the precision, recall, and mAP under CIoU, α-IoU (α = 3), and EIoU loss functions. The results in Table 6 show that the EIoU loss has better overall performance than CIoU and α-IoU (α = 3) losses.

**Figure 11:**
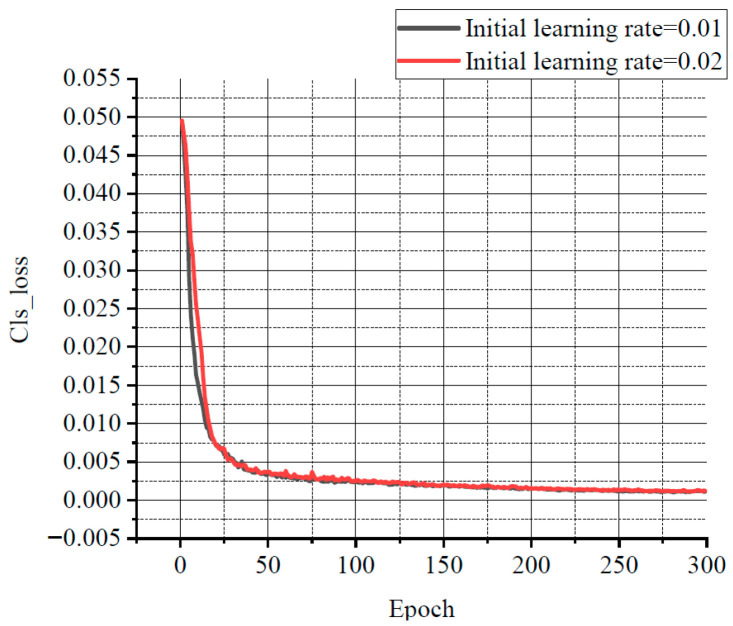
The relationship curve between Cls loss and epoch.

**Figure 12:**
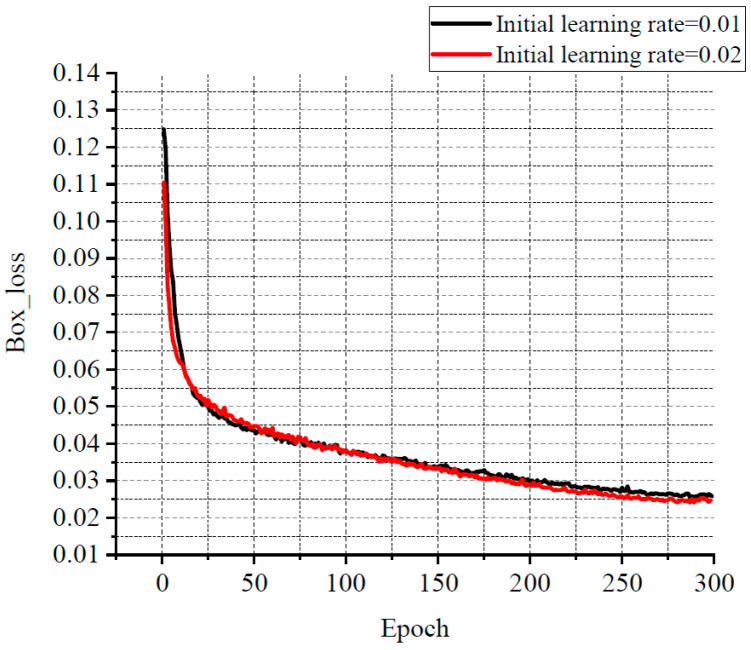
The relationship curve between Box loss and epoch.

**Figure 13:**
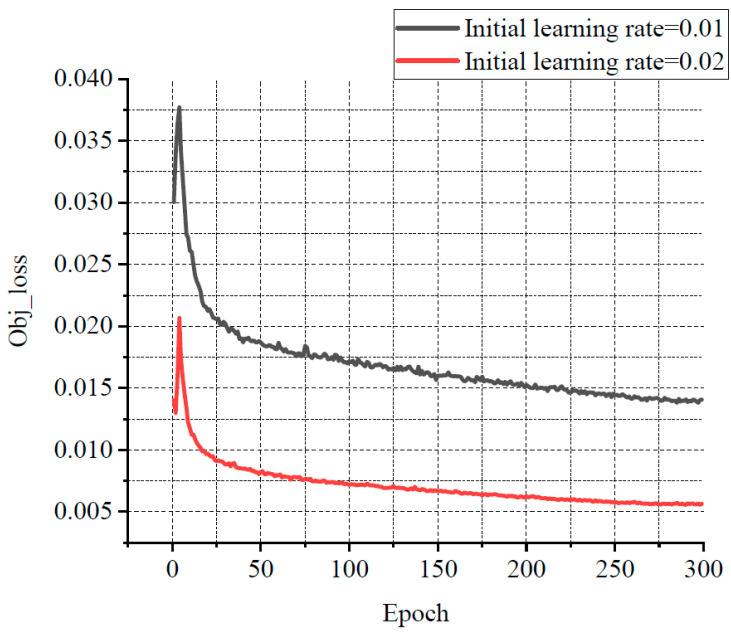
The relationship curve between Obj loss and epoch.

For balancing resource allocation and training time, the relationship among several losses, various learning rates, and the number of iterations was compared through the experiments. Some results are shown in Figure 11, Figure 12 and Figure 13. Classification loss, marked as Cls loss, was used to calculate whether an anchor frame correctly corresponded to the label classification. Position loss, marked as Box loss, was used to calculate the error between the prediction box and calibration box. Confidence loss, marked as Obj_loss, was used to calculate the degree of confidence in a network.

Learning rate is an important hyper parameter in a deep learning network. The batch size affects the generalisation performance of networks. Generally, when the batch size is increased, the learning rate should also be increased according to the linear scaling rule to ensure that the updated weights are equal when using the same sample. In a certain range, increasing the batch size is conducive to the stability of convergence, and a similar effect of learning rate attenuation can be achieved by increasing batch size [28]. Furthermore, with the same number of epochs, the larger the batch size, the fewer the batches required, and the training time is reduced [29]. Therefore, the learning rate in this experiment was set to 0.02 with a decay rate of 0.3, and the final learning rate was 0.006. According to the change of the losses with the number of iterations, the number of iterations was set to 300. The batch size was set to 33. In order to avoid missing small defects as much as possible, the resolution of the input image was adjusted to 960 × 960.

#### 4.5.2. Attention Mechanism

To assess the effectiveness of applying the CA attention mechanism in the LDLFModel, we conducted experimental comparisons with the SE and CBAM attention mechanisms, applying each to the same position within the model. The results in Table 7 indicate that the CA attention mechanism has better comprehensive performance than the SE and CBAM attention mechanisms.

Additionally, CA modules were applied to different positions of the LDLFModel, as shown in Figure 1, to evaluate their impact on precision, recall and mAP. The experimental results in Table 8 display that positions 1, 2, 3, 4, and 5 achieved the highest precision, recall, and mAP. These findings demonstrate that incorporating the CA attention mechanism at these positions significantly enhances detection performance in the LDLFModel.

#### 4.5.3. The Image-Cutting Layer

To assess the effect of the image-cutting layer, we compared detection performance before and after its application. Table 9 shows that precision remained consistently high, with minimal variation, though certain defect types showed marked improvement. For instance, the “mouse bites” defect saw a 0.7% increase in precision. More notably, recall improved visibly post-cutting, particularly for defects such as missing holes, open circuits, short circuits, and spurious copper, with increases of 2.4%, 2.3%, 0.7%, and 9%, respectively. Additionally, the mAP for spurious copper increased by 2.9%. These results indicate that the image-cutting layer positively impacts performance, particularly in improving recall for specific defect types.

**Figure 14:**
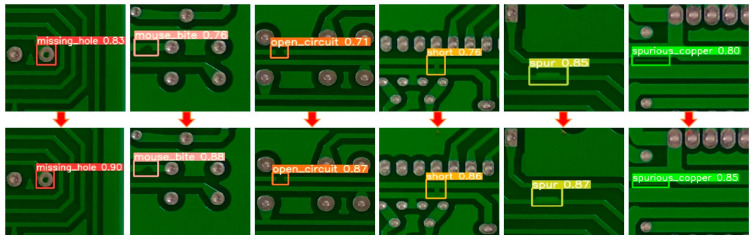
Comparison of six types of defect detection results before and after cutting.

Figure 14 illustrates the comparison of detection performance for six types of defects on the same image, both before and after cutting. The left side of each arrow represents the detection results before cutting, while the right side shows the results after cutting. As can be seen in the figure, the detection accuracy for each defect type improves to some extent following the cutting process. These results confirm that data cutting enhances the detection of high-resolution PCB defects.

## 5. Conclusions

To address the issues of missed detection in small PCB defects while reducing computational complexity and the number of parameters, the LDLFModel is proposed for PCB defect detection. To begin with, the LDLFModel employs the lightweight MobileNet v3 Small-CA with a location attention mechanism as the backbone network. Then, the feature maps are weighted and the location attention mechanism is added to the multi-scale feature fusion module. Moreover, the target frame regression loss function adopts EIoU-loss, which makes the prediction on frame localisation more accurate. Finally, adding a image-cutting layer in the detection module avoids the issue of missed small defect detection on PCB images. A thorough comparative test was conducted using the LDLFModel, Faster R-CNN, EfficientDet, SSD, and YOLO v7 on a publicly available PCB dataset. Based on the experimental results, the LDLFModel increases mAP by 27.45%, 3.7%, 12.1%, and 7.72% compared to Faster R-CNN, EfficientDet, SSD, and YOLO v7, respectively. The model size, computational cost, and the number of parameters of the LDLFModel are much smaller than those of YOLO v7. At the same time, the LDLFModel has a high accuracy on all six types of defects, and the additional image-cutting layer can effectively reduce the issue of misdetection and missed detection of tiny defects. These results suggest that the LDLFModel effectively reduces the model size, the number of parameters, and computational cost compared to the YOLO v7 model, while maintaining strong detection accuracy. Additionally, it shows promise in improving the detection of tiny defects. These improvements suggest that the LDLFModel offers potential advantages for PCB defect detection.

In industrial inspection, it is essential to detect not only surface defects on PCBs but also potential internal issues. The method proposed in this paper is limited to surface defect detection and would need to be integrated with other techniques for identifying internal defects. Additionally, the sliding window approach used to cut the training dataset is conducted offline, which is not conducive to achieving end-to-end integrated processing. As the PCB industry advances, the requirements for defect detection are becoming more stringent. Therefore, ongoing research is necessary to optimize detection methods and improve overall applicability.

## Figures and Tables

**Table 1:** The augmented dataset.

Defect Types	Number of Images
Missing hole	1560
Open circuit	1944
Mouse bite	1728
Spur	1848
Short circuit	1968
Spurious copper	1752

**Table 2:** Comparison of precision, recall, mAP, computational cost, etc.

Backbone	Precision	Recall	mAP	Number of Parameters	Computational Cost	Size of Model
PP-LCNet	96.0%	92.5%	95.3%	4.2 M	9.8 G	9.8 MB
ShuffleNet-v2	97.6%	92.4%	94.9%	5.8 M	8.5 G	9.2 MB
Efficient lite	98.0%	97.0%	98.6%	6.2 M	11.9 G	12.5 MB
MobileNet v3 Small	**98.7%**	**95.4%**	**97.4%**	**3.6 M**	**6.3 G**	**7.4 MB**

**Table 3:** Comparison on different epochs.

Epochs	Precision	Recall	mAP
100	98.84%	96.71%	98.03%
200	99.07%	97.44%	98.74%
300	**99.39%**	**97.57%**	**98.99%**
350	99.39%	97.56%	98.98%

**Table 4:** Comparison of several network models.

Network Model	Model Size	Parameter Number	Inference Time
HOG [2]	0.3 MB	0.04 M	201.0 ms
SIFT [3]	1.3 MB	0.42 M	800.2 ms
YOLO v7	74.8 MB	36.49 M	18.3 ms
MobileNet v3 Small & YOLO v7	10.4 MB	7.2 M	13.5 ms
C3F-SimAM-YOLO [20]	11.4 MB	5.5 M	13.2 ms
SCF-YOLO [21]	9.7 MB	6.1 M	13.0 ms
YOLOv8-PCB [22]	5.6 MB	5.9 M	12.9 ms
LDLFModel	5.4 MB	5.6 M	12.7 ms

**Table 5:** Comparison of computational cost, precision, recall, and mAP.

Network Model	Computational Cost	Precision	Recall	mAP
HOG	13.9 G	60.4%	58.3%	60.1%
SIFT	40.8 G	67.4%	61.6%	66.3%
YOLO v7	16.4 G	93.6%	93.2%	94.2%
C3F-SimAM-YOLO	12.6 G	98.0%	95.4%	97.5%
SCF-YOLO	3.4 G	98.7%	96.3%	98.3%
YOLOv8-PCB	7.1 G	94.7%	94.00%	96.1%
LDLFModel	1.6 G	99.4%	97.6%	99.0%

**Table 6:** Comparison of precision, recall, and mAP.

Model	Precision	Recall	mAP
MobileNet v3 Small + CIoU	98.7%	95.4%	97.4%
MobileNet v3 Small + α-IoU (α = 3)	95.2%	82.5%	88.7%
MobileNet v3 Small + EIoU	98.92%	96.12%	97.62%

**Table 7:** Comparison of different attention mechanisms.

Attention Mechanism	Precision	Recall	mAP
SE	97.5%	97.9%	98.8%
CBAM	99.3%	97.6%	99.0%
CA	99.4%	97.6%	99.0%

**Table 8:** Comparison of different positions of the CA model.

Location of CA Model	Precision	Recall	mAP
1	98.3%	93.4%	95.5%
1, 2, 3	96.4%	87.6%	92.9%
1, 2, 3, 4	94.0%	89.6%	92.7%
2, 3, 4, 5	97.3%	93.3%	96.0%
1, 2, 3, 4, 5	99.4%	97.6%	99.0%

**Table 9:** Comparison of precision, recall, and mAP before and after cutting (%).

Defect Class	Before Cutting			After Cutting		
	Precision	Recall	mAP	Precision	Recall	mAP
Missing holes	99.8	99.3	97.6	100.0	99.6	99.5
Mouse bites	98.8	99.5	99.9	99.8	99.3	99.6
Open circuits	99.4	99.4	97.6	100.0	99.5	99.6
Short circuits	99.1	98.7	97.6	99.0	99.4	99.4
Spurious copper	99.7	99.6	97.6	99.5	96.6	99.5
Spurs	99.5	99.0	97.6	99.5	99.5	99.2

## Data Availability

The PCB defect dataset analysed during the current study are available in the repository of Open Lab on Human Robot Interaction at https://robotics.pkusz.edu.cn/resources/dataset/ (accessed on 6 January 2024), and can also be directly accessed at https://pan.baidu.com/s/1hoPNd7_SAxOWa2XbBZZuTg (accessed on 6 January 2024).
